# Metabolites Analysis on Water-Holding Capacity in Beef *Longissimus lumborum* Muscle during Postmortem Aging

**DOI:** 10.3390/metabo12030242

**Published:** 2022-03-13

**Authors:** Huixin Zuo, Pengsen Wang, Zonglin Guo, Xin Luo, Yimin Zhang, Yanwei Mao

**Affiliations:** 1 Laboratory of Beef Processing and Quality Control, College of Food Science and Engineering, Shandong Agricultural University, Tai’an 271018, China; hxzuo@sdau.edu.cn (H.Z.); sdauwps@163.com (P.W.); luoxin@sdau.edu.cn (X.L.); ymzhang@sdau.edu.cn (Y.Z.); 2 National R&D Center for Beef Processing Technology, Tai’an 271018, China; 3 College of Food Science and Engineering, Gansu Agricultural University, Lanzhou 730070, China; 18394181573@163.com

**Keywords:** beef, aging, metabolome, water-holding capacity, flavor

## Abstract

Currently, the metabolomic research on water-holding capacity (WHC) of beef during postmortem aging is still insufficient. In this paper, the kit method was adopted for energy metabolites testing, the ultra-high-performance liquid chromatography (UHPLC) system was used for sample separation, and the mass spectrometer was applied to collect the primary and secondary spectra of the samples. The results showed that lactic acid reached saturation at day 2 postmortem, while energy metabolites changed significantly within day 2 postmortem (*p* < 0.05). Based on these findings, it was suggested that the energy metabolism qualities of the beef had already achieved a largely stable state at around day 2 postmortem. Then, through metabolomic analysis, 25 compounds were differentially abundant at days 0, 0.5, 1, and 2 during postmortem aging. Within the period of day 0–2 postmortem, the purine metabolism in beef was relatively active until 0.5 d postmortem, while glycolysis metabolism remained active until day 2 postmortem. The functions of the identified metabolites contribute to a more detailed molecular view of the processes behind WHC and are a valuable resource for future investigations into the flavor of postmortem beef.

## 1. Introduction

Water-holding capacity (WHC) is the ability of meat to hold its native and added moisture during fabrication, processing, and storage [1,2]. WHC can largely determine the yield and rate of production. Among all metabolic pathways, glycolysis and its related pathways are the ones with the greatest impact on the WHC of beef during postmortem aging [3,4]. Mohammed et al. [5] conducted a proteomic investigation on beef quality and concluded that the down-regulated expression of glycolysis-reactive protein led to a decrease in anaerobic metabolism and lactic acid production, which in turn affected the pH decline rate, final pH value, and meat color. Surinder et al. [6] further demonstrated that in the absence of sufficient glycogen before slaughter, glycogen had a significant role in the rise and fall of ultimate pH (pH_u_). Since the postmortem pH changes are inextricably related to the lactic acid produced through the glycolysis metabolic pathway, while pH has a strong effect on the WHC of the meat, an investigation into glycogenolysis and glycolysis metabolic pathway is of great importance for understanding the water-holding mechanism of postmortem beef [7,8]. Therefore, the metabolomic analysis of postmortem beef can help realize a proper prediction of the WHC [9].

In addition to metabolic pathways producing metabolites that can directly determine the WHC of postmortem beef, there are also metabolic pathways that can indirectly affect the quality of postmortem beef. According to Poul et al.’s research [10] on the content of muscle glycogen, to prevent the occurrence of dark, firm, and dry (DFD) meat, the minimum glycogen content required for the normal postmortem pH decline from 7.2 to 5.6 was about 53 mmol/kg muscle. Therefore, metabolic pathways that can lead to changes in glycogen contents can also indirectly affect postmortem beef quality. As pointed out by Frank et al. [8], the contents of glycogenolytic enzymes and ionsine monophosphate (IMP)/adenosine monophosphate (AMP) enzymes in DFD beef were lower than those in normal beef. In addition, glutamate and IMP have been identified as flavor-enhancing agents for beef [11], which serve as precursors to the development of beef flavor substances. Therefore, the IMP metabolic pathway has an equally important effect on the flavor variations of postmortem beef. The research findings of Jia et al. [12] demonstrated the key effect of aerobic metabolism on the production of DFD beef. The up-regulated expression of many aerobic metabolism proteins and their related pathways were attributed to the increased adenosine triphosphate (ATP) production in the postmortem muscle [13]. The metabolic pathway of amino acids can create an effect on other metabolic pathways by affecting the synthesis of various enzymes including glycogenolysis and glycolysis pathways, and thus indirectly affecting the WHC of postmortem meat [6,14,15].

Some of the metabolic pathways in postmortem beef would generate metabolites that serve as the flavor precursors of the cooked meat, and purine metabolism is a major pathway through which these metabolites are produced. In addition to indicators of purine metabolites, the metabolism of amino acids also produces a variety of amino acids that are related to the ultimate flavor of beef [16]. Among them, leucine, isoleucine, valine, tyrosine, phenylalanine, hypoxanthine, and inosine are presumably associated with the bitterness of meat, while phenylalanine, alanine, tyrosine, and lactic acid can lead to the development of a sour taste in postmortem beef [11,17,18]. Apart from these, methionine, glutamate, and IMP were also found to have associations with the tenderness of postmortem beef, while the contents of creatine and carnosine can affect the flavor and juiciness of the beef [11,19,20,21,22]. Therefore, indicators acquired from the metabolomic analysis of postmortem beef through the measurement of purine and amino acid metabolic pathways can be used to determine the flavor of beef with postmortem aging.

In recent years, researchers have introduced metabolomics to the field of modern meat science [23]. Metabolomics, another important branch of systems biology, is a new science exploring metabolic processes [24]. Metabolomics explores the metabolic mechanisms of a cell or biological system by analyzing all small molecule metabolites (molecular weight < 1500 Da) before and after a specific interference with the use of high-throughput detection technologies and multivariate data processing [25]. Located at the very end of the biological information flow, metabolome can directly reflect the ultimate physiological status of biological systems, and therefore is widely applied in various fields of studies. Through the analysis of differential metabolites, researchers can understand the specific metabolic mechanism of a certain substance in the biological system. The use of metabolomics can help reveal some light on the nutrition metabolism mechanism of beef and how the meat quality changes over time during postmortem aging [26]. This research intends to apply untargeted metabolomics analysis to detect the metabolites in the samples with the use of the ultra-high-performance liquid chromatography-quadrupole time-of-flight mass spectrometry (UHPLC-Q-TOF-MS) technique. This research reveals the metabolic changes in beef during the postmortem aging process. The research results are of great significance for the establishment and improvement of the theory of WHC and its application in the meat production industry.

## 2. Results

### 2.1. Changes in the Quality Characteristics of Beef during Postmortem Aging

As shown in Table 1, the pH value of the *Longissimus lumborum* (LL) muscle fell to 5.45 at day 2 postmortem and then rose significantly to 5.55 at day 7 (*p* < 0.05), showing a general pattern of falling first and then rising. The time of postmortem aging had a significant effect on drip loss, which increased significantly from 2.19% to 4.1% in the LL muscle within the period of 0–3 d postmortem (*p* < 0.05) and decreased significantly to 3.42% at 7 d (*p* < 0.05). The result of cooking loss showed a similar trend during postmortem aging. According to the analysis results, a significant decrease in the pH value of the meat was observed due to the production of lactic acid through glycolysis. At 3 d postmortem, along with the accumulation of lactic acid, the H^+^ concentration in the meat increased to reach the isoelectric point of myosin, causing the pH value of the beef to rise again in the later stage of aging. As a result, the drip and cooking loss in the LL muscle showed a general trend of falling first and then rising (*p* < 0.05).

### 2.2. Changes in Energy Metabolism of Beef during Postmortem Aging 

Table 2 shows the changes in the content of muscle glycogen, free glucose, and lactic acid of beef LL muscle during postmortem aging. From Table 2, during the aging process, the content of glycogen in beef dropped rapidly, while the content of lactic acid increased drastically until 2 d postmortem, then the rate of increase started to slow down at day 2 postmortem and gradually stabilized. During the process of postmortem aging, the content of glycogen has a direct effect on the content of free glucose by breaking down the free glucose into lactic acid through glycolysis. Because of this, the content of free glucose was tracked and recorded to better understand the differences in glycolysis. Meanwhile, the content of free glucose in the muscle declined significantly within day 2 (*p* < 0.05). According to the results, changes in the energy metabolism were indeed observed during the process of postmortem aging: the glycogen was broken down into lactic acid through glycolysis, which in turn affected the pH value of the beef. Postmortem energy metabolism might influence meat quality by affecting the glycolysis process, which was mainly concentrated at the early stage of postmortem aging (within days 0–2). 

Table 2 shows the changes in the energy levels of ATP, adenosine diphosphate (ADP), and AMP in the LL muscle of beef during postmortem aging. According to Table 2, with the passage of time and as the ATP gradually decreased, the muscle tissues began to fall apart due to the lack of energy control. At this point, the biological system was in a stress status with strengthened metabolism and increased consumption of ATP. The ATP and ADP levels dropped rapidly, while the AMP level declined slowly. As shown in Table 2, the changes in the ATP, ADP, and AMP levels were mainly concentrated in the early stage of postmortem aging (within days 0–2). It was observed that the process of energy metabolism was mainly concentrated in the early stage of postmortem aging (within days 0–2). Thus, these 4 time points were selected, namely days 0, 0.5, 1, and 2, for a follow-up metabolome analysis.

### 2.3. Principal Component Analysis (PCA) 

As shown in Figure S1A,B, a visual inspection was performed on the total ion current chromatograms of all samples. According to the results, the response intensity and retention time of the chromatographic peaks had largely overlapped, indicating relatively small variations caused by instrument errors during the experiment process. In Figure S1, the horizontal coordinate represents the retention time of each chromatographic peak, and the vertical coordinate represents the peak’s intensity value. The instrumental analysis of all samples was with strong signals, large peak capacity, and high repeatability of retention time, which indicated that all the samples collected were reliable and valid. In fact, we used different methods to evaluate the stability of the instrument. The results all showed that the system is stable, which is the premise of this study. Among these methods, the most intuitive is quality control (QC) testing (Figure S1). In addition, we also measure the correlation of QC samples and the relative standard deviation (RSD) of QC samples. Pearson correlation analysis was performed on QC samples, as shown in Figure S2. A general correlation coefficient greater than 0.9 indicates a good correlation. The experimental results showed that the correlation coefficients between QC samples were all above 0.9, indicating good repeatability of the experiment. In Figure S3, the smaller the RSD of the ion peak abundance of QC samples, the better the stability of the instrument. RSD is an important indicator of data quality. In this experiment, the Peak number with an RSD ≤ 30% in QC samples accounted for more than 80% of the total Peak number in QC samples, as shown in Figure S3. This indicates that the instrumental analysis system has good stability and that the data can be used for subsequent analysis.

**Figure 1:**
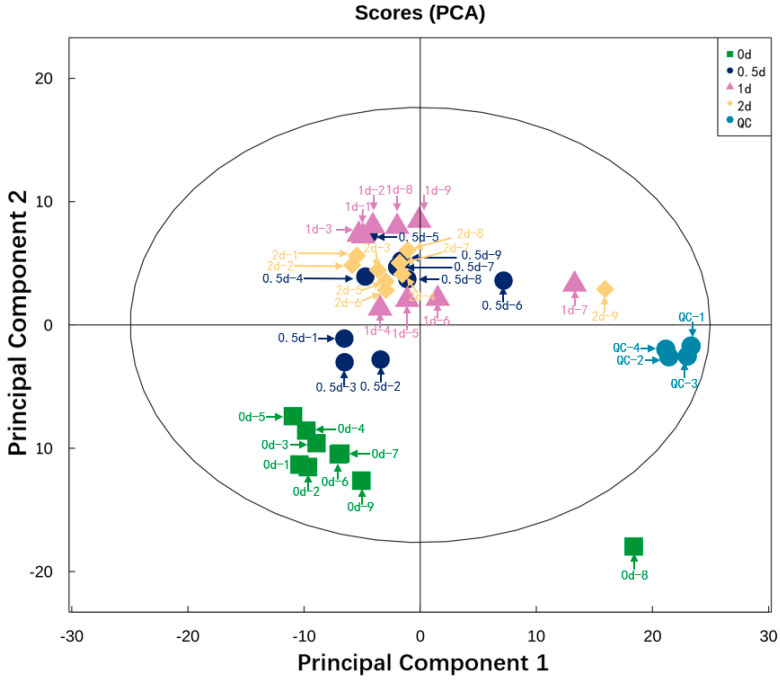
PCA analysis of beef LL muscle during postmortem aging at 4 °C (days 0, 0.5, 1, and 2). PCA is made by all the peak ions. The clustering degree of QC samples reflects the repeatability of the experiment. In the score plot, the green, dark blue, purple, and orange dots represent the sample group at days 0, 0.5, 1, and 2 during postmortem aging, respectively. The blue dots represent the QC samples.

**Figure 2:**
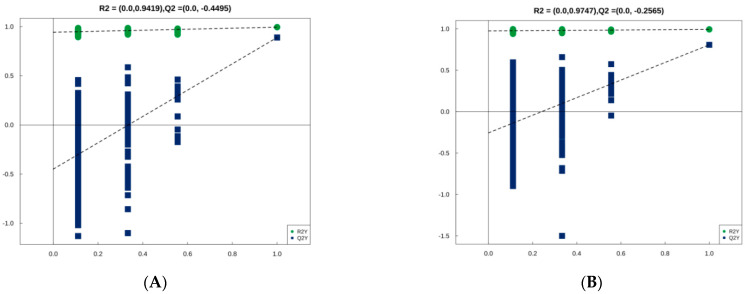
OPLS-DA model. Two hundred rounds of permutation testing were done per component. *p* < 0.05 was used for statistical analysis. (**A**) Positive ion mode; (**B**) Negative ion mode.

Figure 1 shows the result of PCA performed on all the experimental samples. Almost all QC samples were clustered closely, indicating high repeatability of the experiment [27]. Since the dispersion degree of several samples, namely 0 d-8, 0.5 d-6, 1 d-7, 2 d-9, these four samples were deleted. A total of 32 samples (8 samples from day 0, 0.5, 1, and 2, respectively) were conducted for subsequent identification. The degree of dispersion between these sample groups reflects the metabolic differences between the sample groups. It can be observed from the plot that the degree of dispersion between the sample groups was relatively high and the samples were adjacent within the group, which indicates relatively large metabolic differences between the sample groups. Figure 2 shows orthogonal partial least squares discriminant analysis (OPLS-DA) analysis. OPLS-DA can filter out noise irrelevant to classification information and improve the analytical ability and validity of the model. In the OPLS-DA model, the values of R^2^ and Q^2^ were used to assess the goodness of fit and predictive capacity. The closer to 1 they are, the more stable and reliable the model is. R^2^Y represents the rate of model interpretation, and Q^2^Y represents the model predictive ability. We also performed a permutation test to evaluate the goodness of fit of the OPLS-DA model. The model is deemed valid when all Q^2^ and R^2^ values to the left are lower than the original points to the right.

### 2.4. Identification of Differential Metabolites during Postmortem Aging

#### 2.4.1. Identification of Differential Metabolites

The differential metabolites in the LL muscle during postmortem aging at days 0, 0.5, 1, and 2 were identified and screened out. Ions from both positive and negative modes were combined for further analyses. 378 metabolites were identified, with 242 and 202 metabolites identified in positive and negative ion modes, respectively. Generally, metabolites with variable importance in projection (VIP) > 1 were considered to have a significant contribution to model interpretation. In this analysis, “orthogonal partial least squares discriminant analysis (OPLS-DA) VIP > 1” and “*p* < 0.05” were also used as the screening criteria for significantly differential metabolites. Thus, the differential metabolites in the LL muscle during postmortem aging at days 0, 0.5, 1, and 2 were obtained and the significantly differential metabolites are listed in Table 3.

In this research, at days 0.5, 1, and 2 during postmortem aging, 15, 27, and 25 differential metabolites were obtained in the positive ion mode compared with 0 d, while 27, 36, and 34 differential metabolites were obtained in the negative ion mode. The intersection of differential metabolites between groups day 0.5/0, day 1/0, and day 2/0 was taken in positive and negative ion modes, respectively. By comparing the differential metabolites at 0.5 and 0 d, 1 and 0 d, and 2 and 0 d, 10 and 15 intersecting metabolites were obtained in positive and negative ion modes, respectively (Table 3). Thus, a total of 25 differential metabolites were obtained. These metabolites maintained large fold changes throughout the early stage of postmortem aging (day 0–2). The subsequent analysis was centered on these 25 differential metabolites.

#### 2.4.2. Hierarchical Clustering Analysis 

**Figure 3:**
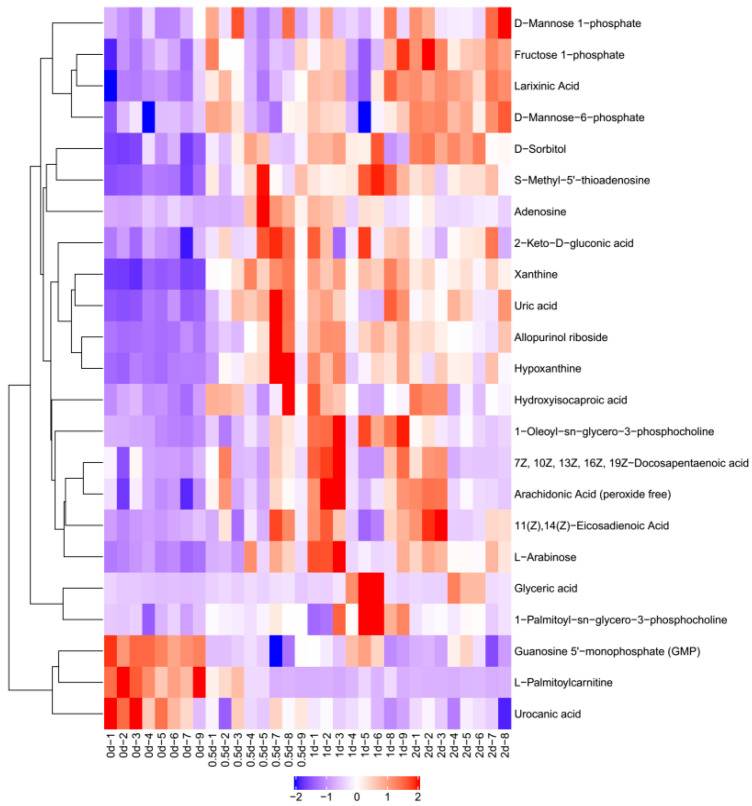
Hierarchical clustering analysis of differential metabolites. A hierarchical cluster analysis was conducted using R software 3.6.3. Through this software, we carried out Z-score standardization operation.

Figure 3 shows the result of hierarchical clustering analysis for significantly differential metabolites (VIP > 1, *p* < 0.05). Metabolites within the same cluster have similar expression patterns. These metabolites are likely to have similar functions, participate in the same metabolic processes, or share the same cellular pathways. In Figure 3, each row represents a differential metabolite (i.e., the vertical coordinate represents the metabolites with significantly differential expression), and each column represents an individual sample group (i.e., the horizontal coordinate represents the sample information). In Figure 2, −2 to 2 represented the color cards, which were automatically generated according to the amount of expression. If the expression level of metabolite is greater, it will be red. Otherwise, it is blue. The shades of the color visually indicate the degree of increase/decrease.

As shown in Table 3 and Figure 3, among the differential metabolites, the expression of L-palmitoylcarnitine, guanosine-5’-monophosphate, and urocanic acid decreased at days 0.5, 1, and 2 compared to day 0 during postmortem aging (*p* < 0.05). All the other differential metabolites saw an increase of varying degrees during postmortem aging. Among them, the expression of S-methyl-5’-thioadenosine, hypoxanthine, allopurinol riboside, xanthine, and uric acid increased by about 4 times.

### 2.5. Kyoto Encyclopedia of Genes and Genomes (KEGG) Pathway Analysis

**Figure 4:**
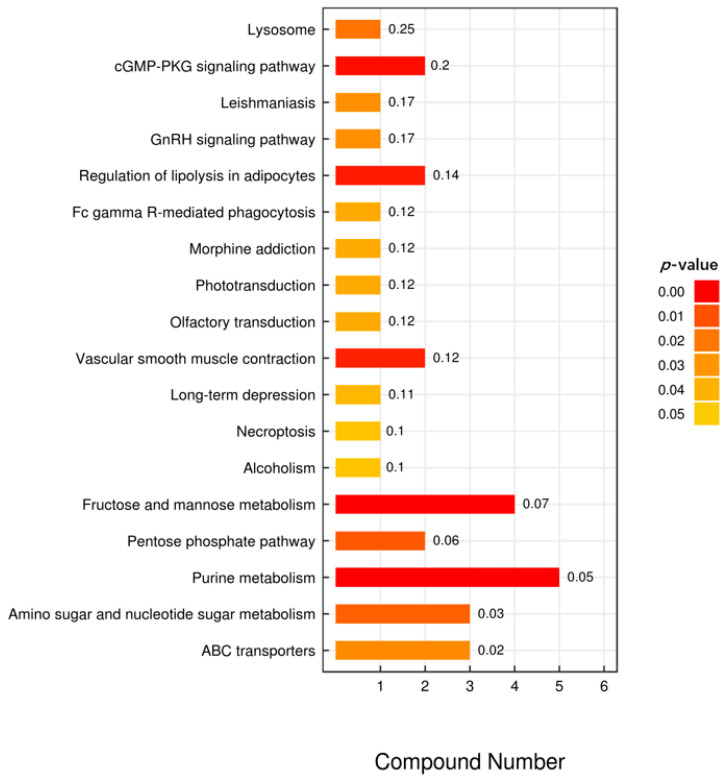
KEGG analysis of intermetabolites. In the bar chart, the vertical axis represents each KEGG metabolic pathway, while the horizontal axis represents the number of differential metabolites contained in each KEGG metabolic pathway and the numbers in front of horizontal bars represent the ratio of differential metabolites to total metabolites. The color represents the *p*-value of the enrichment analysis. The darker the color, the smaller the *p*-value, and the more statistically significant.

According to the KEGG enrichment analysis, the 25 differential metabolites were significantly enriched in 18 pathways. The differential metabolites were involved in a wide range of biological processes, including lysosome (bta04142), fructose and mannose metabolism (bta00051), pentose phosphate pathway (bta00030), purine metabolism (bta00230) and amino sugar, and nucleotide sugar metabolism (bta00520) (Figure 4). These processes may have affected the biological processes in which muscle proteins were involved and in turn affected the WHC of the muscle. 

## 3. Discussion

Due to the termination of the tricarboxylic acid cycle in postmortem muscles and the depletion of the muscular store of creatine phosphate, the supply of ATP, which relies solely on the glycolysis system, would become insufficient [10]. At this point, actin and myosin combine to form actomyosin. As actomyosin starts to accumulate, the muscle gradually stiffens and the WHC of the muscle declines. When glycogen is depleted or the accumulation of acid passivates the glycolytic enzyme system in the carbohydrate metabolism pathways, the glycolysis process can no longer provide energy for the biological system [28]. As a result, actomyosin will be quickly generated in large amounts, leading to a drastic decline in the WHC of the meat [2].

The degree of glycolysis and the accumulation of lactic acid both peri-mortem and postmortem can largely determine the ultimate pH value of the meat. As an important indicator for assessing meat quality, pH value is related to the tenderness, WHC, and color of the meat [12]. Along with a series of complex biochemical reactions taking place in the postmortem muscles, the pH value would go through a series of changes with the passage of time. The aging process of the meat would be negatively affected when the pH value is either too high or too low. Meanwhile, the metabolic processes of certain carbohydrates, e.g., mannose, can affect the glycosylation process of specific proteins and change the ability of carrier proteins in the cell membrane. The pentose phosphate pathway can generate substantial amounts of nicotinamide adenine dinucleotide phosphate-reduced (NADPH) and provide raw materials for other metabolic pathways, thus exerting an indirect effect on the WHC of the muscle [29]. As shown by the experiment results, the content of lactic acid in beef reached a near-saturation state at day 2 postmortem, while ATP, ADP, and AMP contents in the ATP metabolic pathway decreased drastically within day 0–2 postmortem and then stabilized after that, which largely coincided with the accumulation of lactic acid within the same period. Provided a proper postmortem glycolysis process, the meat product can be kept in a low-acid status with a stable pH value. Depending on the muscle type, part, and age of the meat, the pH value of the meat can be kept within the range of 5.4–6.2. Under such circumstances, the isoelectric focusing of muscle proteins can be avoided, allowing some distance between the muscle proteins. As a result, higher WHC can be achieved under the effect of the capillary phenomenon [30].

During the process of postmortem aging, amino acid metabolism also has an indirect impact on multiple metabolic pathways through the generation of various enzymes and histones to maintain the life activities of the muscle as peri-mortem. This would contribute to the postmortem aging of the muscle and improve its WHC [29]. The postmortem metabolism of amino acids can directly change the muscle structure through biochemical activities, such as the protein degradation of muscle by lysosomes. During postmortem aging, certain critical proteins would be degraded by cathepsin and calpain, which damages the original muscle structure. In the meantime, the muscle structure would become loose and more porous, which increases the WHC of the muscle [31].

Purines refer to a group of substances that exist in various animal bodies, mainly in the form of purine nucleotides [32]. Purines are involved in the energy supply, metabolic regulation, and composition of coenzymes in biological systems. Purine metabolism can mainly be divided into anabolism and catabolism [33]. During postmortem aging, xanthine is synthesized from guanine through guanine deaminase, and hypoxanthine is produced through xanthine oxidase [34]. The measured content of xanthine can indicate the freshness of the meat, and the end product of purine metabolism is uric acid [35]. As can be observed, the content of uric acid in beef reached its maximum level at day 0.5 postmortem, which then remained unchanged through the long period after the peak. In addition, the contents of hypoxanthine, allopurinol riboside, and xanthine also reached their highest point by almost the same time at day 0.5 postmortem. This indicated that purine metabolism was highly active within the period of 0–0.5 d postmortem, which then tended to stabilize in the later stage of aging. However, the development of meat flavor is closely related to the presence of flavor precursors, which include free fatty acids, carbohydrates, nucleotides, and free amino acids [36]. In addition to purine metabolism, which was highly active within 0–0.5 d postmortem, the variations of free fatty acids, including arachidonic acid, 11(Z), 14(Z)-eicosatetraenoic acid, and 7Z, 10Z, 13Z, 16Z, 19Z-docosapentaenoic acid, also stabilized within day 0–2 postmortem. Thus, it was inferred that the flavor of beef with postmortem aging had largely stabilized in the early days of storage.

## 4. Materials and Methods

### 4.1. Animals and Sampling

Nine crossbred offspring of *Luxi* cattle and *Simmental* cattle (weight 356–390 kg) were of the same age (18–24 months old) and fed on the same diet in the same batch. The animals were kept off feed before slaughtering but given free access to water. Animals were stunned by captive bolt pistols, and their blood was drained. The LL muscle (the anterior 12th rib to the last lumbar vertebrae) was randomly extracted from a commercial abattoir (Yangxin Yiliyuan Halal Meat Co., Ltd., Binzhou City, Shandong Province, China). Samples were washed with phosphate-buffered saline (PBS) to remove any blood and contaminants on the surface. The experiment was undertaken following the guidelines outlined by the Animal Ethics Committee at Shandong Agricultural University (SDAUA-2021-095, 20210315).

### 4.2. pH

pH was recorded from 45 min (0 d) up to 7 d during postmortem aging with a portable pH meter (SenvenGo, Mettler-Toledo, Switzerland), which was adjusted for each measurement with standard buffer before being inserted into muscles [2].

### 4.3. Drip Loss

A 50-g sample of meat (10 cm × 5 cm × 1 cm) cut perpendicularly to muscle fibers was taken immediately during postmortem aging, suspended in a polyamide/polyethylene bag (55.86 cm^3^/m^2^/24 h oxygen transmission rate, 4.40 g/m^2^/24 h moisture transmission rate) at 4 °C and labeled as day 0. After 48 h, the sample was taken out of the bag, dried on absorbent paper, and reweighed. The percent change in weight over the subsequent 48 h was taken as the drip loss, as described by Honikel [37]. Subsequently, samples were taken every 24 h and marked as days 1, 2, 3, 5, and 7, respectively. Samples taken at 12 h postmortem were labeled as day 0.5.

### 4.4. Cooking Loss

A 50-g sample of meat (10 cm × 5 cm × 1 cm) cut perpendicularly to muscle fibers was taken immediately during postmortem aging, wrapped in an aluminum foil bag and labeled as day 0. Samples were cooked in preheated water until the core temperature reached 70 °C [38]. Sample internal temperature was monitored with a data logger and a thermocouple probe inserted horizontally at the middle of the steak. Cool down to room temperature naturally. The sample was taken out of the bag, dried on an absorbent sheet, and reweighed. Cooking loss was the difference in weight between the precooked weight and blotted dry post-cooked weight, and it was expressed as a percentage of the precooked weight [39]. The sampling time of cooking loss at days 0.5, 1, 2, 3, 5, and 7 was the same as that of drip loss.

### 4.5. Energy Metabolism Indexes

The contents of muscle glycogen, lactic acid, free glucose, ATP, ADP, and AMP were determined by the corresponding reagent kits produced by Nanjing Jiancheng Reagent Co., Ltd.(Nanjing, China). The specific operation and calculation of test results were performed according to the instructions of the reagent kits. 

### 4.6. Extraction of Metabolites

A quantitative analysis of metabolites in the *Longissimus lumborum* was conducted at day 0, 0.5 (12 h), 1, and 2 during postmortem aging under a temperature of 0–4 °C. The samples were taken out and treated with liquid nitrogen grinding. Then, 100 mg of each sample were weighed and placed into 200 μL of pre-cooled water with 800 μL of methanol/acetonitrile (1:1) [40]. The solution was mixed well and sonicated in an ice bath for 60 min, precipitated at −20 °C for 1 h, and then centrifuged at 16,000× *g* for 20 min at 4 °C. The supernatant was extracted and evaporated in a high-speed vacuum centrifuge. During the mass spectrometry, 100 μL of acetonitrile-water solution (1:1) was added and centrifuged at 14,000× *g* for 15 min at 4 °C, and the supernatant was extracted for analysis [41].

### 4.7. UHPLC-QTOF-MS Analysis

The samples were separated on a HILIC column by the Agilent 1290 Infinity LC UHPLC system, as previously reported [42]. The temperature of the column was kept at 25 °C, the flow rate was 0.5 mL/min, and the injection volume was 2 μL. The mobile phase consisted of (A) water + 25 mM ammonium acetate + 25 mM ammonia; and (B) acetonitrile. The gradient elution program was set as follows: 0–0.5 min, 95% (B); 0.5–0.7 min, (B) changed linearly from 95% to 65%; 7–8 min, (B) changed linearly from 65% to 40%; 8–9 min, (B) was maintained at 40%; 9–9.1 min, (B) changed linearly from 40% to 95%; 9.1–12 min, (B) was maintained at 95%. Throughout the analysis process, the samples were placed in a 4  °C auto-sampler. The AB Triple TOF 6600 mass spectrometer was used to collect the primary and secondary spectra of the samples [40]. After this, the differential metabolites were identified and screened out with the help of the database. The result was used to examine the changes in the types and abundance of metabolites in the *Longissimus lumborum* during postmortem aging under the storage condition of 0–4 °C. Then, the metabolites were mapped to the KEGG pathway database to obtain the enrichment results of their metabolic pathways. The change patterns of the metabolites were examined at day 0, 0.5, 1, and 2 during postmortem aging, and the metabolic pathways related to the WHC were analyzed.

### 4.8. Statistical Analysis

Statistical significance was assessed with analysis of variance (ANOVA) using the general linear model (GLM) of SPSS 20.0 (SPSS Inc., Chicago, IL, USA). Experiments adopted a randomized block design. The differences between means were detected using the Student-Newman-Keuls (SNK) test at the 5% significance (*p* < 0.05) level. All the results were represented as the mean value ± standard error. The raw data of mass spectra were converted into the mzML format using ProteoWizard (Version 3.0, Palo Alto, CA, USA). For retention time shift correction and peak analysis (including identification, extraction, integration, and alignment), the XCMS online software (https://xcmsonline.scripps.edu/landing_page.php?pgcontent=mainPage, accessed on 23 January 2022) was used. OSI-SMMS (version 1.0) was used for substance identification with the self-built database. Chroma TOF4.3X (LECO Corporation, St. Joseph, MI, USA) and LECO-Fiehn Rtx5 database (LECO Corporation, St. Joseph, MI, USA) were used for data processing. To further explore the impact of differentially expressed metabolites, enrichment analysis was performed. The KEGG database was used for functional annotation for differential metabolites. The biological pathways of differential metabolites and their interrelations were analyzed using the KEGG database and KEGG orthology (KO) classification system. The KEGG pathway enrichment analyses were applied based on Fisher’s exact test, considering the whole metabolites of each pathway as a background data set.

## 5. Conclusions

This research probed into the activity differences of various energy traits within the period of 0–7 d postmortem, and applied metabolomic analyses to explain the variations in the WHC of beef. Based on the changes of metabolites in beef within the period of day 0–2 postmortem aging, the variations of metabolites in the LL muscle were examined in detail. At days 0.5, 1, and 2 compared with days 0, 15, 27, and 25 differential metabolites were obtained in the positive ion mode, while 27, 36, and 34 differential metabolites were obtained in the negative ion mode, respectively. The content of lactic acid in beef reached a near-saturation state at day 2 postmortem, while at the same time the decrease of ATP content started to slow down, indicating an almost complete stop of glycolysis metabolism at day 2 postmortem. On the other hand, purine metabolism peaked at 0.5 d postmortem, while no obvious changes were observed in the intermediate metabolites, such as xanthine and hypoxanthine, or the final metabolite uric acid after 0.5 d postmortem. According to the changes in the differential metabolites above, we can use them to predict the flavor and WHC of beef. We can also explain the improvement of flavor and other meat quality in beef during postmortem aging. Within day 2 postmortem, intermediate metabolites produced by purine metabolism can provide good flavor for postmortem beef. Meanwhile, with the glycolysis metabolism, the quality of postmortem beef is changing. Since this paper is only concerned with glycolytic metabolism, amino acid metabolism and purine metabolism in postmortem beef, further research can be performed on the role of more metabolic pathways in postmortem beef quality. 

## Figures and Tables

**Table 1:** Changes in quality characteristics of beef LL muscle during postmortem aging at 4 °C (d 0, 0.5, 1, 2, 3, 5, and 7).

Traits	0 d	0.5 d	1 d	2 d	3 d	5 d	7 d
pH	5.75 ± 0.01 ^a^	5.71 ± 0.02 ^b^	5.65 ± 0.03 ^c^	5.45 ± 0.03 ^e^	5.46 ± 0.03 ^e^	5.49 ± 0.01 ^e^	5.55 ± 0.02 ^d^
Drip loss (%)	2.19 ± 0.06 ^f^	2.31 ± 0.04 ^g^	2.49 ± 0.08 ^e^	2.86 ± 0.03 ^d^	4.1 ± 0.03 ^a^	3.91 ± 0.02 ^b^	3.42 ± 0.03 ^c^
Cooking loss (%)	19.15 ± 0.63 ^d^	19.29 ± 0.37 ^d^	19.45 ± 0.82 ^d^	22.21 ± 0.93 ^bc^	25.37 ± 1.18 ^a^	23.02 ± 0.84 ^b^	21.27 ± 0.55 ^c^

^a–^^g^ Means without common superscripts in a row within are different (*p* < 0.05).

**Table 2:** Changes in energy metabolism of beef LL muscle during postmortem aging at 4 °C (d 0, 0.5, 1, 2, 3, 5, and 7).

Traits	0 d	0.5 d	1 d	2 d	3 d	5 d	7 d
Glycogen (mg·g^−1^)	5.58 ± 0.09 ^a^	4.63 ± 0.07 ^b^	3.72 ± 0.07 ^c^	2.19 ± 0.1 ^d^	2.03 ± 0.08 ^de^	1.91 ± 0.09 ^e^	1.89 ± 0.12 ^e^
Lactic acid (ng·mL^−1^)	100.67 ± 11.68 ^c^	118.33 ± 12.50 ^c^	172 ± 11.00 ^b^	231.67 ± 12.10 ^a^	231 ± 12.77 ^a^	225 ± 10.54 ^a^	217 ± 15.39 ^a^
Free glucose (mmol·L^−1^)	9.39 ± 0.07 ^a^	8.33 ± 0.07 ^b^	7.90 ± 0.14 ^c^	6.48 ± 0.1 ^e^	6.39 ± 0.15 ^e^	6.31 ± 0.13 ^e^	6.26 ± 0.11 ^e^
ATP (μmol·g^−1^)	3.53 ± 0.08 ^a^	2.72 ± 0.09 ^b^	1.98 ± 0.11 ^c^	0.77 ± 0.12 ^d^	0.66 ± 0.12 ^d^	0.57 ± 0.11 ^d^	0.51 ± 0.14 ^d^
ADP (μmol·g^−1^)	4.52 ± 0.1 ^a^	2.60 ± 0.09 ^b^	1.36 ± 0.08 ^c^	0.49 ± 0.12 ^d^	0.36 ± 0.11 ^d^	0.30 ± 0.10 ^d^	0.29 ± 0.10 ^d^
AMP (μmol·g^−1^)	0.3 ± 0.03 ^a^	0.20 ± 0.04 ^b^	0.21 ± 0.03 ^b^	0.19 ± 0.03 ^b^	0.15 ± 0.04 ^b^	0.14 ± 0.04 ^b^	0.14 ± 0.01 ^b^

^a–^^e^ Means without common superscripts in a row within are different (*p* < 0.05).

**Table 3:** Identification of differential metabolites in LL muscle during postmortem aging.

Name	Accession Number	Adduct	*m/z*	Fold Change		
				0.5/0 d	1/0 d	2/0 d
***Positive ion mode***						
S-Methyl-5’-thioadenosine	M298T94	(M + H)^+^	298.0968	3.77	4.70	3.69
L-Palmitoylcarnitine	M400T164	(M + H)^+^	400.3411	0.29	0.10	0.08
1-Palmitoyl-sn-glycero-3-phosphocholine	M496T162	(M + H)^+^	496.3374	1.50	2.31	1.43
2-Keto-D-gluconic acid	M159T220	(M + H − 2H_2_O)^+^	159.0265	2.71	2.60	2.18
Hypoxanthine	M137T185	(M + H)^+^	137.0456	3.97	3.96	3.26
Larixinic acid	M127T467	(M + H)^+^	127.0382	1.40	1.63	1.94
Allopurinol riboside	M269T211	(M + H) ^+^	269.0875	3.66	4.24	3.25
1-Oleoyl-sn-glycero-3-phosphocholine	M522T148	(M + H)^+^	522.3536	1.43	2.65	1.52
D-Mannose-6-phosphate	M261T494	(M + H)^+^	261.0360	1.50	1.48	2.12
Adenosine	M250T91	(M + H − H_2_O)^+^	250.0923	4.65	3.33	2.23
***Negative ion mode***						
Xanthine	M151T213_2	(M − H)^−^	151.0262	4.19	4.41	3.63
Guanosine 5’-monophosphate	M362T455	(M − H)^−^	362.0479	0.70	0.79	0.72
S-Methyl-5’-thioadenosine	M296T91	(M − H)^−^	296.0794	3.78	3.88	4.58
Uric acid	M167T193	(M − H)^−^	167.0198	4.95	4.69	4.27
Hypoxanthine	M135T191_2	(M − H)^−^	135.0309	3.03	3.06	2.84
D-Sorbitol	M181T285	(M − H)^−^	181.0710	2.59	3.08	3.77
Glyceric acid	M105T296	(M − H)^−^	105.0188	2.43	12.85	6.99
L-Arabinose	M149T150	(M − H)^−^	149.0445	1.95	2.68	2.37
Hydroxyisocaproic acid	M131T133	(M − H)^−^	131.0703	2.46	2.13	2.23
Arachidonic acid	M303T38	(M − H)^−^	303.2318	1.92	2.70	2.29
Urocanic acid	M154T266	(M + NH_4_ − 2H)^−^	154.0611	0.66	0.58	0.54
Fructose 1-phosphate	M519T448	(2M − H)^−^	519.0505	1.73	1.95	2.74
11(Z),14(Z)-Eicosadienoic acid	M307T38	(M − H)^−^	307.2615	1.73	1.90	2.21
7Z, 10Z, 13Z, 16Z, 19Z-Docosapentaenoic acid	M329T38	(M − H)^−^	329.2466	1.58	2.29	1.61
D-Mannose 1-phosphate	M259T467	(M − H)^−^	259.0219	1.41	0.70	1.74

Fold change means the ratio of changes in metabolite abundance between 0.5 and 0 d, 1 and 0 d, and 2 and 0 d, respectively.

## Data Availability

The data presented in this study are available from the corresponding author on request.

## Supplementary Materials

The following supporting information can be downloaded at: https://www.mdpi.com/article/10.3390/metabo12030242/s1, Figure S1. Overlapping spectra of total ion current of quality control (QC) samples. (A) Positive ion mode; (B) Negative ion mode. Figure S2. Correlation map of QC samples. (A) Positive ion mode; (B) Negative ion mode. Figure S3. The relative standard deviation (RSD) for QC samples. (A) Positive ion mode; (B) Negative ion mode.

## References

[1] Huang C., Hou C., Ijaz M., Yan T., Li X., Li Y., Zhang D. (2020). Proteomics discovery of protein biomarkers linked to meat quality traits in post-mortem muscles: Current trends and future prospects: A review. Trends Food Sci. Tech..

[2] Zuo H., Han L., Yu Q., Niu K., Zhao S., Shi H. (2016). Proteome changes on water-holding capacity of yak *Longissimus lumborum* during postmortem aging. Meat Sci..

[3] Setyabrata D., Cooper B.R., Sobreira T.J.P., Legako J.F., Martini S., Kim Y.H.B. (2021). Elucidating mechanisms involved in flavor generation of dry-aged beef loins using metabolomics approach. Food Res. Int..

[4] Hollung K., Veiseth E., Frøystein T., Aass L., Langsrud Ø., Hildrum K.I. (2007). Variation in the response to manipulation of post-mortem glycolysis in beef muscles by low-voltage electrical stimulation and conditioning temperature. Meat Sci..

[5] Mohammed G., Robyn D.W., Peter P., Ranjith R., Anne M.M., Maria L.-P., Daniel F., José M.L., Igor T., Brigitte P. (2021). Dark-cutting beef: A brief review and an integromics meta-analysis at the proteome level to decipher the underlying pathways. Meat Sci..

[6] Surinder S.C., Eric M.E. (2018). Postmortem glycolysis and glycogenolysis: Insights from species comparisons. Meat Sci..

[7] Ribeiro F.A., Lau S.K., Furbeck R.A., Herrera N.J., Henriott M.L., Bland N.A., Fernando S.C., Subbiah J., Sullivan G.A., Calkins C.R. (2021). Ultimate pH effects on dry-aged beef quality. Meat Sci..

[8] Frank K., Steven D.H., Janet R., Deborah L.V., Gretchen G.M., Ranjith R. (2021). Changes in glycolytic and mitochondrial protein profiles regulates postmortem muscle acidification and oxygen consumption in dark-cutting beef. J. Proteomics.

[9] Osorio M.T., Moloney A.P., Brennan L., Monahan F.J. (2012). Authentication of beef production systems using a metabolomic-based approach. Animal.

[10] Poul H., Anders K., Mogens T.J., Niels O., Jette S.P. (2002). Metabolic conditions in porcine *Longissimus* muscle immediately pre-slaughter and its influence on peri- and post-mortem energy metabolism. Meat Sci..

[11] Dashdorj D., Amna T., Hwang I. (2015). Influence of specific taste-active components on meat flavor as affected by intrinsic and extrinsic factors: An overview. Eur. Food Res. Technol..

[12] Jia X., Hildrum K.I., Westad F., Kummen E., Aass L., Hollung K. (2006). Changes in enzymes associated with energy metabolism during the early post-mortem period in *Longissimus thoracis* bovine muscle analyzed by proteomics. J. Proteome Res..

[13] Tao Y., Ma L., Li D., Tian Y., Liu J., Liu D. (2021). Proteomics analysis to investigate the effect of oxidized protein on meat color and water holding capacity in Tan mutton under low temperature storage. LWT-Food Sci. Technol..

[14] Gagaoua M., Hughes J., Terlouw E.M.C., Warner R.D., Purslow P.P., Lorenzo J.M., Picard B. (2020). Proteomic biomarkers of beef colour. Trends Food Sci. Tech..

[15] Ijaz M., Li X., Zhang D., Hussain Z., Ren C., Bai Y., Zheng X. (2020). Association between meat color of DFD beef and other quality attributes. Meat Sci..

[16] Greta B., Franziska W., Nino T., Edwin J., Volker H., Andreas J., Monika G. (2021). Analysis of aging type- and aging time-related changes in the polar fraction of metabolome of beef by ^1^H NMR spectroscopy. Food Chem..

[17] Koutsidis G., Elmore J.S., Oruna-Concha M.J., Campo M.M., Wood J.D., Mottram D.S. (2008). Water-soluble precursors of beef flavour. Part II: Effect of post-mortem conditioning. Meat Sci..

[18] Pomponio L., Bukh C., Ruiz-Carrascal J. (2018). Proteolysis in pork loins during superchilling and regular chilling storage. Meat Sci..

[19] Kodani Y., Miyakawa T., Komatsu T., Tanokura M. (2017). NMR-based metabolomics for simultaneously evaluating multiple determinants of primary beef quality in Japanese Black cattle. Sci. Rep..

[20] Macy R.L., Naumann H.D., Bailey M.E. (1970). Water-soluble flavor and odor precursors of meat. 5. Influence of heating on acid-extractable non-nucleotide chemical constituents of beef, lamb and pork. J. Food Sci..

[21] Purchas R.W., Rutherfurd S.M., Pearce P.D., Vather R., Wilkinson B.H.P. (2004). Concentrations in beef and lamb of taurine, carnosine, coenzyme Q_10_, and creatine. Meat Sci..

[22] Koutsidis G., Elmore J.S., Oruna-Concha M.J., Campo M.M., Wood J.D., Mottram D.S. (2008). Water-soluble precursors of beef flavour: I. Effect of diet and breed. Meat Sci..

[23] Wedekind R., Keski-Rahkonen P., Robinot N., Mercier F., Engel E., Huybrechts I., Scalbert A. (2020). Metabolic signatures of 10 processed and non-processed meat products after in vitro digestion. Metabolites.

[24] Man K.Y., Chan C.O., Tang H.H., Dong N., Capozzi F., Wong K.H., Kwok K.W.H., Chan H.M., Mok D.K.W. (2021). Mass spectrometry-based untargeted metabolomics approach for differentiation of beef of different geographic origins. Food Chem..

[25] Liu T., Mo Q., Wei J., Zhao M., Tang J., Feng F. (2021). Mass spectrometry-based metabolomics to reveal chicken meat improvements by medium-chain monoglycerides supplementation: Taste, fresh meat quality, and composition. Food Chem..

[26] Lee S.M., Kwon G.Y., Kim K.O., Kim Y.S. (2011). Metabolomic approach for determination of key volatile compounds related to beef flavor in glutathione-Maillard reaction products. Anal. Chim. Acta..

[27] Theodoridis G., Pechlivanis A., Thomaidis N.S., Spyros A., Georgiou C.A., Albanis T., Skoufos I., Kalogiannis S., Tsangaris G.T., Stasinakis A.S. (2021). Metabolic signatures of 10 processed and non-processed meat products after in vitro digestion. Metabolites.

[28] Huff Lonergan E., Zhang W., Lonergan S.M. (2010). Biochemistry of postmortem muscle—Lessons on mechanisms of meat tenderization. Meat Sci..

[29] Serra X., Guerrero L., Guardia M.D., Gil M., Sañudo C., Panea B., Campo M.M., Olleta J.L., García-Cachán M.D., Piedrafita J. (2008). Eating quality of young bulls from three Spanish beef breed-production systems and its relationships with chemical and instrumental meat quality. Meat Sci..

[30] Paredi G., Raboni S., Bendixen E., de Almeida A.M., Mozzarelli A. (2012). “Muscle to meat” molecular events and technological transformations: The proteomics insight. J. Proteomics.

[31] Farouk M.M., Mustafa N.M., Wu G., Krsinic G. (2012). The “sponge effect” hypothesis: An alternative explanation of the improvement in the waterholding capacity of meat with ageing. Meat Sci..

[32] Dewulf J.P., Marie S., Nassogne M.C. (2021). Disorders of purine biosynthesis metabolism. Mol. Genet. Metab..

[33] Zheng M., Huang Y., Ji J., Xiao S., Ma J., Huang L. (2018). Effects of breeds, tissues and genders on purine contents in pork and the relationships between purine content and other meat quality traits. Meat Sci..

[34] Chandra S.P., Rooma D. (2014). Biosensing methods for xanthine determination: A review. Enzyme Microb. Tech..

[35] Yazdanparast S., Benvidi A., Abbasi S., Rezaeinasab M. (2019). Enzyme-based ultrasensitive electrochemical biosensor using poly(l-aspartic acid)/mwcnt bio-nanocomposite for xanthine detection: A meat freshness marker. Microchem. J..

[36] Mottram D.S. (1998). Flavour formation in meat and meat products: A review. Food Chem..

[37] Honikel K.O. (1998). Reference methods for the assessment of physical characteristics of meat. Meat Sci..

[38] Taniguchi M., Arakawa A., Nishio M., Okamura T., Ohnishi C., Kadowaki K., Kohira K., Homma F., Matsumoto K., Ishii K. (2020). Differential metabolomics profiles identified by CE-TOFMS between high and low intramuscular fat amount in fattening pigs. Metabolites.

[39] Zuo H., Han L., Yu Q., Guo Z., Ma J., Li M., La H., Han G. (2018). Proteomic and bioinformatic analysis of proteins on cooking loss in yak *Longissimus thoracis*. Eur. Food Res. Technol..

[40] Gao Y., Li J., Li X., Li X., Yang S., Chen N., Li L., Zhang L. (2020). Tetrahydroxy stilbene glycoside attenuates acetaminophen-induced hepatotoxicity by UHPLC-Q-TOF/MS-based metabolomics and multivariate data analysis. J. Cell Physiol..

[41] Yang S., Wang X., Duan C., Zhang J. (2020). A novel approach combining metabolomics and molecular pharmacology to study the effect of Gei Herba on mouse hematopoietic function. Biomed. Pharmacother..

[42] Hu L., Che L., Wu C., Curtasu M.V., Wu F., Fang Z., Lin Y., Xu S., Feng B., Li J. (2019). Metabolomic profiling reveals the difference on reproductive performance between high and low lactational weight loss sows. Metabolites.

