# Supplementary material for: Metabolites Analysis on Water-Holding Capacity in Beef Longissimus lumborum Muscle during Postmortem Aging
Source: Metabolites. 2022 Mar 13;12(3):242. doi: 10.3390/metabo12030242 (PMC8950885; doi:10.3390/metabo12030242)
Supplement: Supplementary file 1 [file metabolites-12-00242-s001.zip › metabolites-1589065-supplementary.pdf]

Supplementary Figures

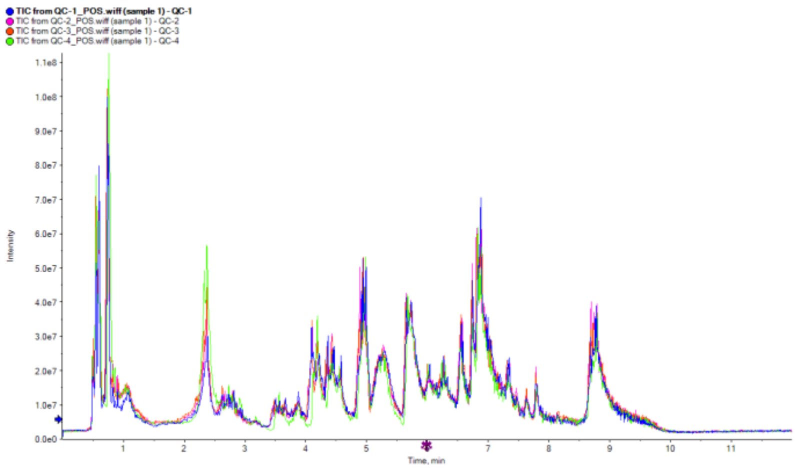

(A)

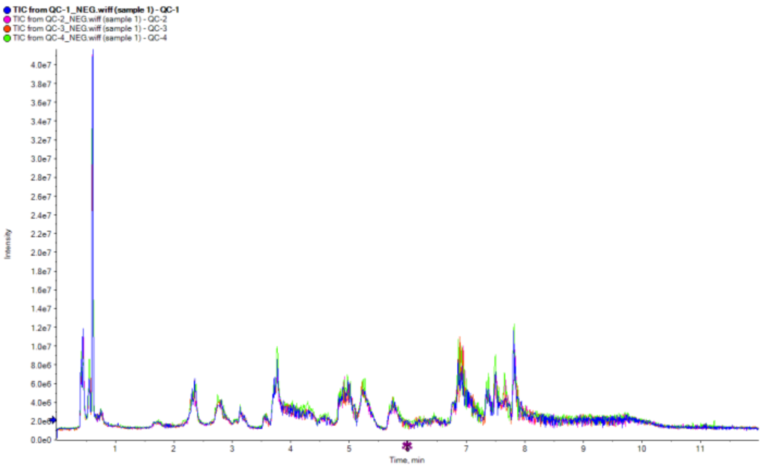

(B)

Figure S1. Overlapping spectra of total ion current of quality control (QC) samples. (A) Positive ion mode; (B) Negative ion mode.

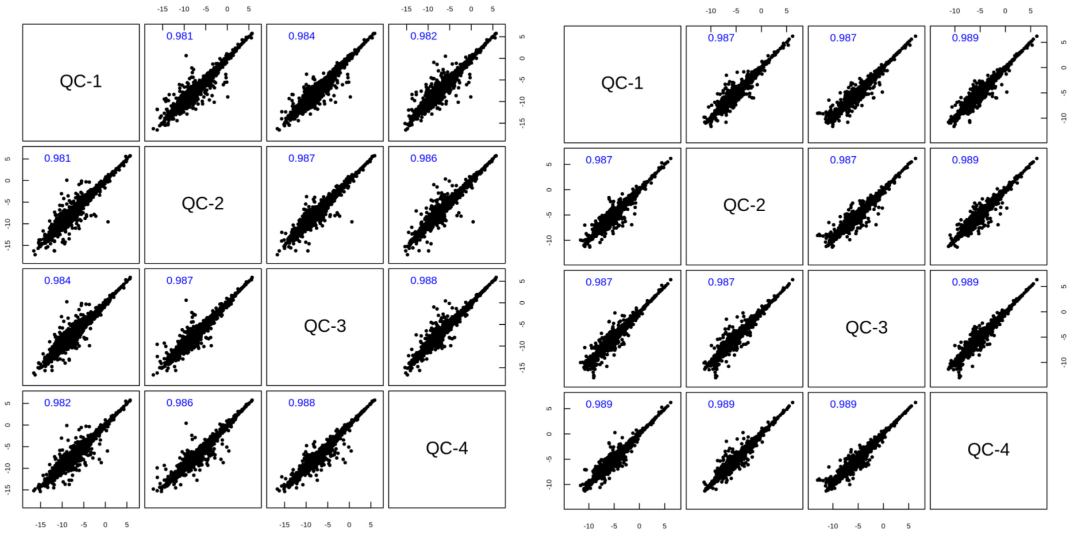

(A) (B)

Figure S2. Correlation map of QC samples. (A) Positive ion mode; (B) Negative ion mode.

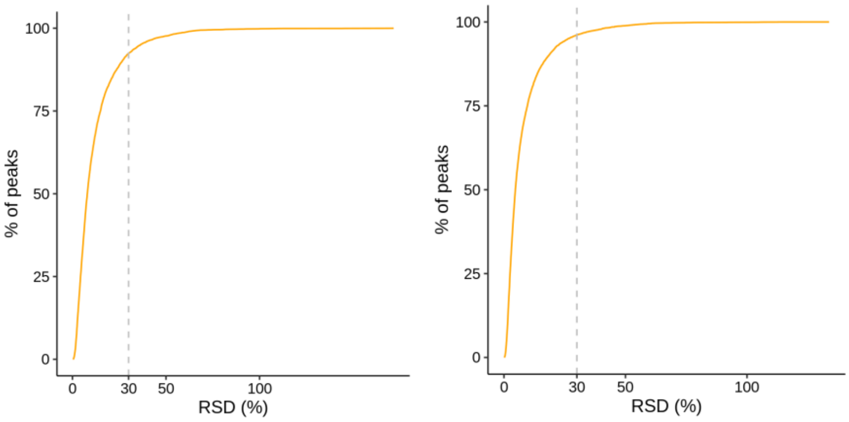

(A) (B)

Figure S3. The relative standard deviation (RSD) for QC samples. (A) Positive ion mode; (B) Negative ion mode.
